# Dominant Suppression of β1 Integrin by Ectopic CD98-ICD Inhibits Hepatocellular Carcinoma Progression

**DOI:** 10.3390/ijms17111882

**Published:** 2016-11-10

**Authors:** Bo Wu, Yang Zhou, Yu Wang, Xiang-Min Yang, Zhen-Yu Liu, Jiang-Hua Li, Fei Feng, Zhi-Nan Chen, Jian-Li Jiang

**Affiliations:** 1 National Translational Science Center for Molecular Medicine, Cell Engineering Research Centre and Department of Cell Biology, State Key Laboratory of Cancer Biology, Fourth Military Medical University, Xi’an 710032, China; soldier2158wubo@163.com (B.W.); 15209262969@163.com (Y.Z.); yxiangmind@163.com (X.-M.Y.); qqqzhenyu20052008@163.com (Z.-Y.L.); m18729251936@163.com (J.-H.L.); fengfei122@126.com (F.F.); 2 Department of Oncology, State Key Discipline of Cell Biology, Xijing Hospital, Fourth Military Medical University, Xi’an 710032, China; wangyufmmu@163.com; 3 Institute of Stomatology, Chinese People’s Liberation Army General Hospital, Beijing 100853, China

**Keywords:** CD98 heavy chain, carcinoma, hepatocellular, disease progression, integrins, protein transport

## Abstract

Hepatocellular carcinoma (HCC) is currently the third most common cause of cancer-related death in the Asia-Pacific region. Our previous work showed that knockdown of CD98 significantly inhibits malignant HCC cell phenotypes in vitro and in vivo. The level of CD98 in the membrane is tightly regulated to mediate complex processes associated with cell–cell communication and intracellular signaling. In addition, the intracellular domain of CD98 (CD98-ICD) seems to be of vital importance for recycling CD98 to the membrane after it is endocytosed. The intracellular and transmembrane domains of CD98 associate with β-integrins (primarily β1 but also β3), and this association is essential for CD98 mediation of integrin-like signaling and complements dominant suppression of β1-integrin. We speculated that isolated CD98-ICD would similarly suppress β1-integrin activation and inhibit the malignant behaviors of cancer cells. In particular, the exact role of CD98-ICD has not been studied independently in HCC. In this study, we found that ectopic expression of CD98-ICD inhibited the malignant phenotypes of HCC cells, and the mechanism possibly involves β1-integrin suppression. Moreover, the expression levels of CD98, β1-integrin-A (the activated form of β1-integrin) and Ki-67 were significantly increased in HCC tissues relative to those of normal liver tissues. Therefore, our preliminary study indicates that ectopic CD98-ICD has an inhibitory role in the malignant development of HCC, and shows that CD98-ICD acts as a dominant negative mutant of CD98 that attenuates β1-integrin activation. CD98-ICD may emerge as a promising candidate for antitumor treatment.

## 1. Introduction

Hepatocellular carcinoma (HCC) is one of the most aggressive tumors with a poor therapeutic outcome after combined surgical treatment, radiotherapy and chemotherapy [1,2,3]. CD98, a heterodimeric transmembrane protein complex comprising a glycosylated heavy chain (SLC3A2) and a non-glycosylated light chain, and it is recognized as a negative prognostic marker for several human tumors including HCC [4,5]. The extracellular domain of CD98 interacts with its light chains, which are essential for the transport of amino acids [6,7,8]. Intracellular and transmembrane domains of CD98 associate with β-integrins (mainly β1 but also β3) to mediate integrin-like signaling [9,10].

Initially, overexpression of isolated integrin β1 cytoplasmic domains blocks integrin activation, and CD98 rescues this suppression [11]. While regulated activation of integrins is critical for tissue homeostasis, overexpressed β1-integrin detected in several types of human cancers indicated poor prognosis, and it is associated with metastasis and chemo-resistance of cancer cells [12,13,14]. Indeed, integrin-dependent events, such as survival, proliferation, migration and even malignant transformation can be activated by CD98 without integrin ligation or extracellular matrix engagement [10,15,16,17,18]. Recently, CD98 was reported to enable matrix assembly and support RhoA-driven contractility, and it contributed to carcinogenesis by amplifying a positive feedback loop, which increases both extracellular matrix stiffness and resulting cellular responses [15].

Dysregulated endocytosis influences the surface protein level directly and significantly contributes to several hallmarks of cancer [19,20]. Our previous work indicated that CD98 and CD147 are internalized through Arf6 (ADP-ribosylation factor 6)-related CIE (clathrin-independent endocytosis) in HCC cells [21]. In particular, the acidic amino acid clusters in the cytoplasmic tails of CD98 and CD147 are of great importance for sorting these proteins to the tubular endosomes arranged along microtubules [22,23]. Moreover, the intracellular domain, which is exposed to the cytoplasm and interacts with potential cellular sorting apparatus, seems of vital importance for de novo synthesized protein targeting to the membrane. Despite the fundamental role of the intracellular domain of CD98 (CD98-ICD), it has not been independently studied previously. As CD98 complements the dominant suppression of integrin, and the transmembrane and cytoplasmic domains of CD98 are necessary and sufficient for interactions with β1-integrin, we speculate that isolated CD98-ICD would similarly suppress β1-integrin activation and inhibit the malignant behaviors of cancer cells.

Here, we report that over-expression of CD98-ICD inhibited the malignant phenotypes of HCC cells at least by suppressing β1-integrin activation. Additionally, we demonstrate that the expression levels of CD98, β1-integrin-A (activated form of β1-integrin) and Ki-67 were significantly increased in HCC tissues relative to those of normal liver tissues, and these three biomarkers were concomitantly overexpressed in HCC tissues. Clinical association analyses indicated that the expression of CD98, β1-integrin-A and Ki-67 was associated with clinical pathological characteristics of HCC patients including tumor size and tumor stage. Therefore, CD98-ICD may be of potential value to HCC treatment.

## 2. Result

### 2.1. CD98-ICD Inhibits HCC Cell Adhesion, Spreading and Proliferation

**Figure 1:**
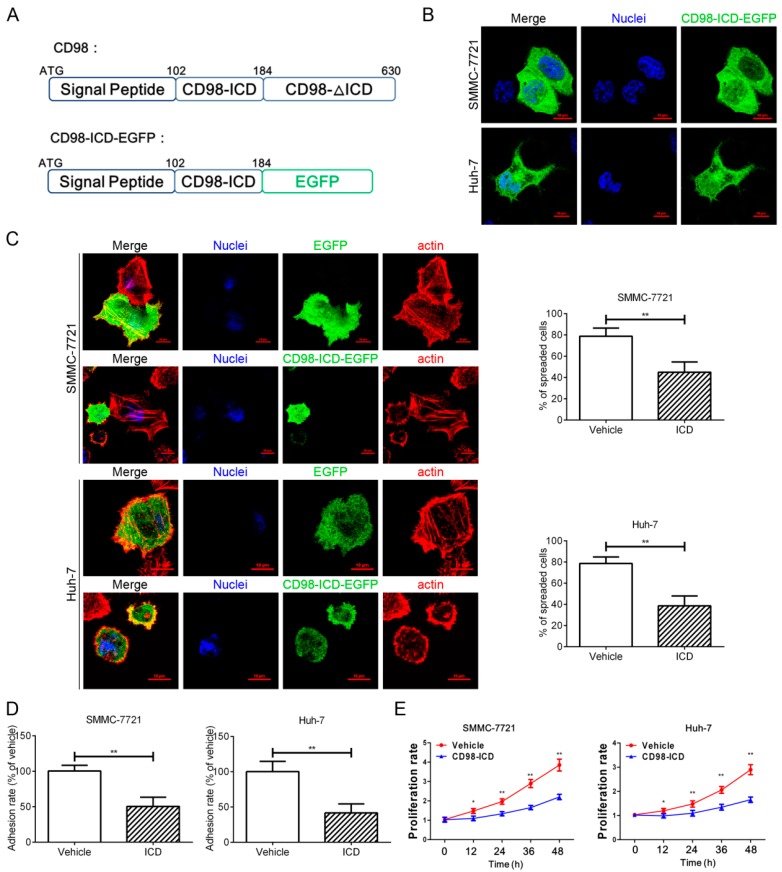
CD98-ICD inhibits SMMC-7721 and Huh-7 cell adhesion, spreading and proliferation in vitro. (**A**) Schematic illustration of CD98 and CD98-ICD-EGFP. CD98 is a type II transmembrane protein, and the N-terminal is located in the cytoplasm. The N-terminal signal peptide and intracellular domain of CD98 (CD98-ICD) were fused with EGFP to create CD98-ICD-EGFP; (**B**) representative distribution of CD98-ICD-EGFP in SMMC-7721 and Huh-7 cell lines. SMMC-7721 (5 × 10^4^) or Huh-7 cells (5 × 10^4^) were cultured on coverslips and transfected with 2 μg of CD98-ICD-EGFP for 36 h. The distribution of CD98-ICD-EGFP (green) was visualized with a confocal microscope. Nuclei: blue. Bar, 10 µm; (**C**) spreading assay of SMMC-7721 and Huh-7 (Vehicle or ICD) cells. The indicated transfected cells (5 × 10^4^) were cultured on coverslips with 1% Matrigel for 2 h, and then the cells were fixed and stained with rhodamine-phalloidin (red) for visualization. CD98-ICD-EGFP: green; Nuclei: blue. The percentage of spread cells was determined by scoring >100 cells. The data are the mean of the “% of spread cells ± SD” from three independent experiments. Bar, 10 µm. ** *p* < 0.01; (**D**) adhesion ability assay. The graphed adhesion rate to the extra cellular matrix as described previously (*n* = 3); (**E**) the effects of CD98-ICD on the cell proliferation rate of SMMC-7721 (or Huh-7) cells were detected by staining with WST-1 (water soluble tetrazolium) for the indicated time. The graphs show mean ± SD of three independent experiments. * *p* < 0.05 and ** *p* < 0.01.

To investigate the role of CD98-ICD in the progression of HCC, a vector encoding the signal peptide and intracellular domain of CD98 was constructed (CD98-ICD-EGFP, Figure 1A). As expected, lacking the extracellular and transmembrane domains, most of the exogenous CD98-ICD-EGFP (signal peptide and intracellular domain of CD98 fused with EGFP) localized to the cytoplasm in SMMC-7721 and Huh-7 cells (Figure 1B). After transfection with CD98-ICD-EGFP, the spreading and adhesion ability of SMMC-7721 and Huh-7 cells was significantly decreased (Figure 1C,D). Moreover, both SMMC-7721 and Huh-7 cells transfected with CD98-ICD-EGFP exhibited a lower proliferation rate compared to that of control cells (Figure 1E). The above results indicated that CD98-ICD inhibits adhesion, spreading and proliferation of both SMMC-7721 and Huh-7 cells.

### 2.2. CD98-ICD Inhibits Tumorigenicity of SMMC-7721 Cells

**Figure 2:**
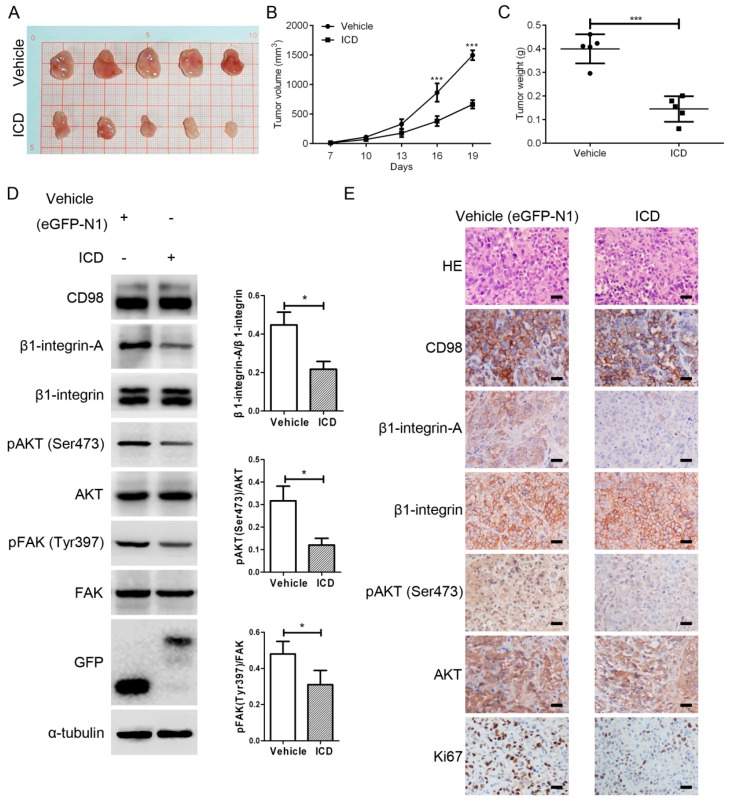
CD98-ICD inhibits tumorigenicity of SMMC-7721 cells in vivo. (**A**,**C**) Primary tumors were harvested and weighed (each group had five animals, and experiments were repeated twice, each round or square dot represent one tumor). The data are expressed as mean ± SD; *** *p* < 0.001; (**B**) tumor volumes were determined at various time points; *** *p* < 0.001; (**D**) expression levels of β1-integrin-A, p-Akt and p-FAK were analyzed by Western blot. Western blot scanning densitometry is shown on the right for three independent experiments on the right. Blots were probed for β1-integrin, AKT, or FAK independently to ensure equal protein loading; * *p* < 0.05; (**E**) expression levels of β1-integrin-A, p-Akt and p-FAK analyzed by immunohistochemistry. Scale bar, 100 μm.

To observe the effect of CD98-ICD on tumorigenicity in vivo, we first constructed SMMC-7721 cells stably transfected with EGFP-N1 or CD98-ICD-EGFP. Mice were then subcutaneously implanted with 107 cells. Tumor volumes were determined every three days starting on Day 7 (Figure 2B). Mice were sacrificed and the primary tumors were harvested and weighed (Figure 2A,C). The weights of the excised tumors in the control group were significantly more than those in the ICD group (*p* < 0.001). Western blot analyses showed that the expression of β1-integrin-A, p-Akt (phosphorylated protein kinase B) and p-FAK (phosphorylated focal adhesion kinase) was decreased by CD98-ICD overexpression (Figure 2D), and the decreased expression of β1-integrin-A and p-Akt were further confirmed by immunohistochemical examination of xenograft tumor sections (Figure 2E). Immunohistochemical analysis also showed that the cell proliferation marker Ki-67 was downregulated by exogenous CD98-ICD. The in vivo tumorigenicity experiment further confirmed the suppressive role of CD98-ICD in SMMC-7721 cells. Taken together, these results suggest that CD98-ICD inhibits the malignant behaviors of HCC cells.

### 2.3. Membrane CD98 Was Not Influenced by Exogenous CD98-ICD

**Figure 3:**
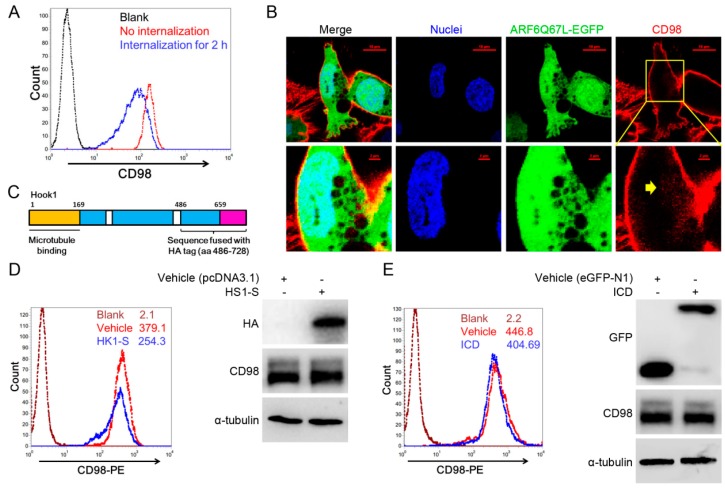
CD98-ICD was not directly involved in the recycling process of CD98. (**A**) Internalization of CD98. Subconfluent SMMC-7721 cells were incubated with anti-CD98 at 4 °C for 10 min, and then unbound antibodies were washed from the cells. Then, cells were allowed to internalize the surface label at 37 °C for 0–120 min. Bound antibodies were then labeled with Dylight 488 conjugated goat-anti-mouse secondary antibody and analyzed by FACS; (**B**) representative confocol microscopy images of CD98 (red) in SMMC-7721 cells transfected with ARF6Q67L–EGFP (green). Vacuolar membranes induced by ARF6Q67L: yellow arrow; CD98: red; Nuclei: blue. Bar, 10 µm; (**C**) schematic representation of the domain organization of hook1 (the amino terminus is shown in yellow; aa 1–168, coiled-coil region in blue; aa 659–728, carboxyl terminus in pink). HK1-S encoding for aa 486–728 of hook1 was fused with an HA tag and used in subsequent experiments; (**D**,**E**) membrane CD98 was determined by FACS after SMMC-7721 cells were transfected with HK1-S or CD98-ICD for 36 h. The mean fluorescence intensity (MFI) of each group is indicated beside the histograms. The data shown are representative of three individual experiments.

Typically, CD98 is distributed on the cell membrane, and the membrane CD98 can be internalized through an ARF6-related CIE pathway to maintain cellular homeostasis. Here, CD98 internalization was assayed with a surface labeling strategy. Quantitation by FACS (fluorescence activated cell sorting) revealed that approximately one third of the labeled CD98 was internalized into the cytoplasm in 2 h (Figure 3A). Figure 3B shows that transfection of ARF6Q67L-EGFP, a constitutively activated form of ARF6, induced the formation of special structures termed vacuolar membranes, and CD98 (red) accumulated in the vacuolar membranes (yellow arrow). After internalization, CD98 can recycle back to the membrane through the mediation of hook1. As a microtubule- and cargo-tethering protein, hook1 recognizes the cytoplasmic tail of CD147 to aid in sorting CD147 and CD98 into Rab22a-dependent tubules associated with recycling [22]. We then cloned HK1-S (the cargo-tethering portion of hook1) (Figure 3C), which inhibits CD147 recycling competitively. After transfection of HK1-S into SMMC-7721 cells, the FACS results showed that membrane CD98 was decreased, while Western blot showed that the total CD98 level did not change (Figure 3D). Therefore, CD98 may be arrested in the cytoplasm after recycling of the protein was competitively blocked. However, CD98-ICD-EGFP only slightly inhibited the membrane expression of CD98 (Figure 3E). Therefore, even though internalized CD98 could be recycled, the CD98-ICD likely does not directly participate in the CD98 recycling process to inhibit HCC progression.

### 2.4. CD98-ICD Inhibits β1-Integrin Signaling

**Figure 4:**
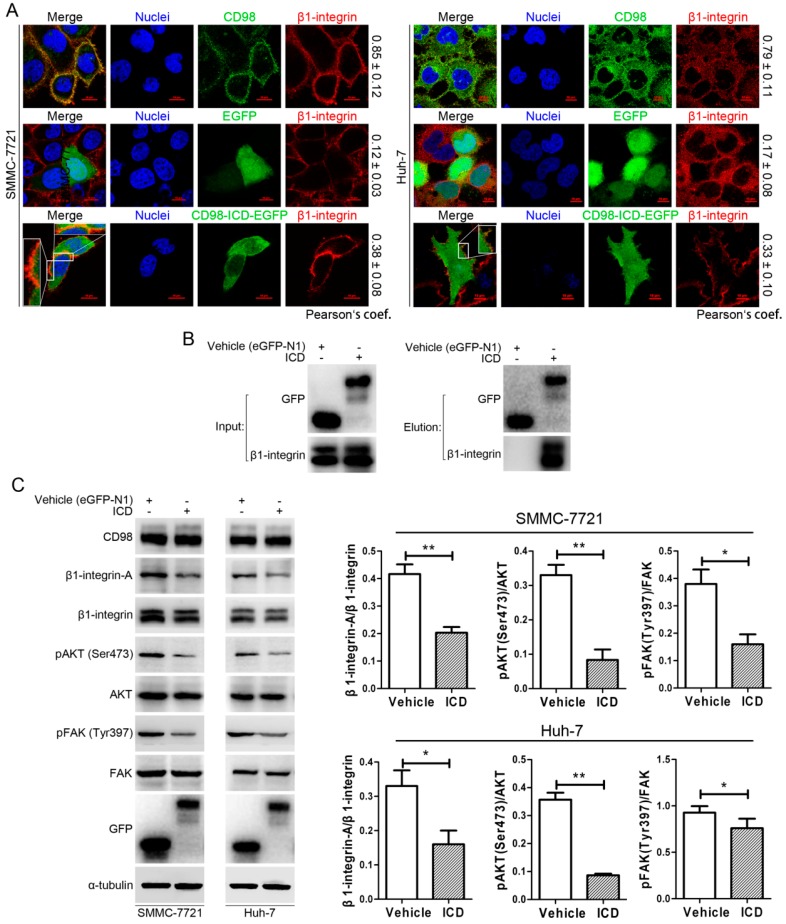
CD98-ICD inhibits β1-integrin signaling. (**A**) Co-localization of β1-integrin and CD98-ICD in SMMC-7721 and Huh-7 cells. The co-localization of β1-integrin (red) and CD98 (green) was first analyzed as a positive control (**upper** panel). Then, after cells were transfected with eGFP-N1 (**middle** panel) or CD98-ICD-EGFP (**lower** panel) for 36 h, β1-integrin was visualized to analyze co-localization with CD98-ICD. (Pearson’s coefficient is indicated as numerical data on the right of each panel, *n* > 3). Nuclei: blue. Bar, 10 µm; (**B**) Co-IP analyses of β1-integrin and CD98-ICD interaction in SMMC-7721 cells; (**C**) changes in the molecular expression patterns were detected in SMMC-7721 and Huh-7 cells transfected with EGFP-N1 or CD98-ICD-EGFP. Western blot scanning densitometry for three independent experiments is shown on the right. Blots were probed for β1-integrin, AKT, or FAK independently to ensure equal protein loading. * *p* < 0.05, ** *p* < 0.01.

CD98 is a single-pass type II transmembrane protein that mediates integrin-dependent signaling. As shown in the upper panel of Figure 4A, CD98 had a good co-localization with β1-integrin in SMMC-7721 and Huh-7 cells. In the lower panel of Figure 4A, exogenous CD98-ICD-EGFP had a better co-localization with β1-integrin compared to EGFP (middle panel). Moreover, co-IP showed that CD98-ICD-EGFP could interact with β1-integrin (Figure 4B). As CD98 is required for efficient adhesion-induced activation of integrin-dependent signals, such as AKT, major contributors to integrin-dependent signaling, including pAKT and pFAK, were decreased in SMMC-7721 and Huh-7 cells transfected with CD98-ICD compared with their corresponding vehicle groups (Figure 4C). These data indicated that exogenous CD98-ICD could interact with β1-integrin and inhibit downstream integrin signaling.

### 2.5. Immunohistochemical Detection of the Expression of CD98, β1-Integrin-A and Ki-67 in HCC Specimens

**Figure 5:**
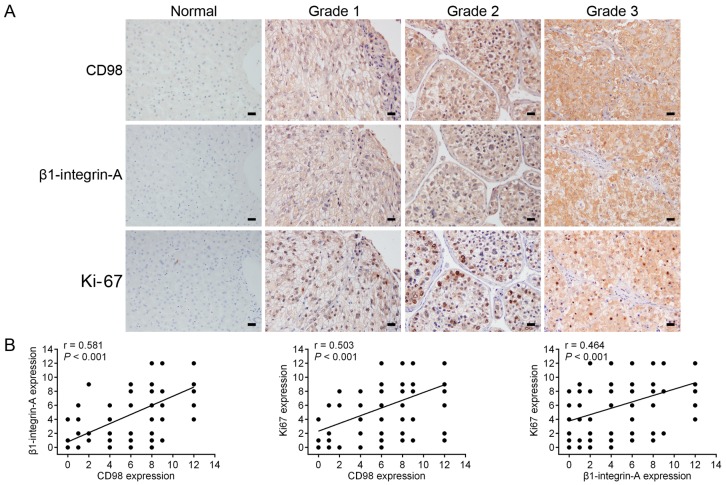

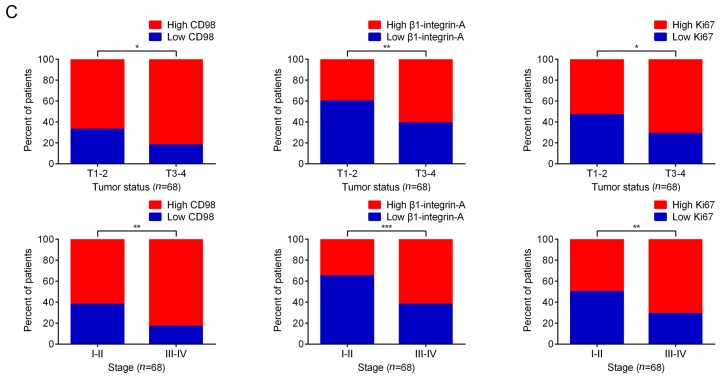
Coordinated expression of CD98, β1-integrin-A and Ki-67 in HCC patient specimens determined by immunohistochemical analysis. (**A**) Representative images of immunohistochemical staining of CD98, β1-integrin-A and Ki-67 using consecutive tissue sections from same HCC patients (Scale bars, 100 μm); (**B**) scatter plot analysis of the correlation between protein expression levels of CD98, β1-integrin-A and Ki-67 in 68 HCC tissues. The dots on the graphs represent more than one specimen; (**C**) the expression of CD98, β1-integrin-A and Ki-67 was positively correlated with tumor size and disease stage (* *p* < 0.05, ** *p* < 0.01, *** *p* < 0.001).

To explore the role of CD98, β1-integrin-A and Ki-67 in HCC tumorigenesis, the expression status in a total of sixty-eight pairs of human HCC specimens and adjacent non-cancerous specimens was characterized by immunohistochemical staining. As shown in Figure 5A, the expression of CD98, β1-integrin-A and Ki-67 were confirmed to be higher in human HCC specimens than in adjacent non-cancerous specimens. Moreover, Spearman rank correlation analysis showed significant positive correlations between CD98 and β1-integrin-A, CD98 and Ki-67, and β1-integrin-A and Ki-67 protein levels (Figure 5B). We next sought to determine whether the expression levels of CD98, β1-integrin-A and Ki-67 were associated with the pathological progression of HCC. As shown in Figure 5C, the expression levels of CD98, β1-integrin-A and Ki-67 were significantly increased in HCC samples from stage T3–4 patients, compared to the levels in HCC samples from stage T1–2 patients. The expression levels of CD98, β1-integrin-A and Ki-67 were significantly increased in HCC samples from stage III–IV patients, compared to the levels in HCC samples from stage I–II patients. Taken together, these results revealed that the expression levels of CD98, β1-integrin-A and Ki-67 were upregulated and correlated with cancer progression and malignancy in HCC.

## 3. Discussion

This study underscored the inhibition of HCC progression in vitro and in vivo by ectopically expressed CD98-ICD through dominant suppression of β1-integrin. The work also demonstrated that CD98, β1-integrin-A and Ki-67 were upregulated in HCC tissues, and the concomitant expression of these three biomarkers was associated with clinical pathological characteristics of HCC patients including tumor size and tumor stage. Considering the underlying molecular mechanisms might be opening promising avenues for HCC, CD98-ICD could be an effective candidate for further development of anti-cancer drugs.

CD98 was first identified and reported as an early T-cell activation antigen that facilitates lymphocyte clonal expansion and enables adaptive immunity [4,24]. By amplifying integrin signals, CD98 prevented apoptosis while promoted proliferation of lymphocytes. However, these integrin-dependent signals can also provoke cancer development and rapid proliferation of tumor cells [4,25]. Integrins are a family of membrane proteins found only in animals, and they have been implicated in two major types of activities: adhesion of cells to their substratum (or to other cells) and transmission of signals between the external environment and the cell interior. CD98 acts as a “molecular facilitator” in the plasma membrane, and transmembrane and cytoplasmic domains of CD98, are necessary and sufficient for interactions with β1-integrin [16,26,27]. Using chimeras of CD98 and the type II membrane protein CD69, Henderson et al. showed that the transmembrane domain of CD98 is necessary and sufficient for integrin association in cells. Moreover, aa 82–87 in the putative cytoplasmic/transmembrane region of CD98 appeared to be critical for the oncogenic potential of the molecule [26]. However, here we found that exogenous CD98-ICD had an inhibitory role in HCC progression (Figure 1 and Figure 2), which appears contradictory to the cancer-promoting role of CD98. More confusing, Hara et al. demonstrated that truncation of the extracellular domain of CD98 enhanced tumorigenicity in NIH3T3 cells [28].

To decipher this paradoxical role of CD98, we first concentrated on its vesicle recycle characteristics. Derailed endocytosis and thereafter subsequent aberrant distribution of membrane proteins, including adhesion molecules, conjunction proteins and many receptor tyrosine kinases, could can create cancerous cell behavior in normal cells [29,30]. CD98 can be internalized through an ARF6-related CIE pathway and recycled back to the membrane through the mediation of hook1, Rab22a and microtubules [22,23]. CD98 was constitutively internalized (Figure 3A) and accumulated in vacuolar membranes after transfection with ARF6Q67L (a constitutively activated form), indicating the conservation of CD98 internalization in HCC cell lines (Figure 3B). Overexpression of HK1-S but not CD98-ICD competitively inhibits CD98 membrane localization (actually a recycling process) in the experiment. Therefore, our results indicated that CD98-ICD may not be directly involved in CD98 recycling. However, CD98 recycling may be mediated through the intracellular domain of CD147, with CD147 binding CD98 at the extracellular domain.

As discussed above and reported previously, CD98 constitutively associates with β1-integrin and rescues its suppression [11]. As a speculative possibility, CD98-ICD may associate with β1-integrin. Clearly, both CD98 and β1-integrin are co-localized on the membrane of SMMC-7721 and Huh-7 cells with a good co-localization coefficient. Interestingly, exogenous CD98-ICD was prone to localize to the membrane, and had better co-localization with β1-integrin compared to control EGFP, implying that CD98-ICD interacts with β1-integrin to some extent (Figure 4A). Co-IP analysis further confirmed the interaction between CD98-ICD and β1-integrin (Figure 4B). Most integrins normally exist on the cell surface in an inactive conformation. Conformational alterations of the cytoplasmic domains could can be propagated through the molecule rapidly, increasing the integrin’s affinity for an extracellular ligand, therefore activate activating the integrins. The cytoplasmic domains of integrins bind a wide array of proteins, including talin. Mutations in talin that block interaction with β integrin subunits also prevent activation of integrins and adhesion to the extracellular matrix [31,32]. Here, a similar mechanism is applied for exogenous CD98-ICD suppression of the β1-integrin activation (Figure 4C). Recently, ubiquitination of the intracellular domain of CD98 by MARCH8 was shown to limit cell proliferation and clonal expansion [25]. This is another study, to some extent, underlines the inhibitory role of CD98 activity. Moreover, syndecan-1 cCTF (cytoplasmic C-terminal fragment) antagonizes syndecan-1 dependent tumor cell migration by competing with the full length syndecan-1 for intracellular interaction partners and thereby reduces signaling of syndecan-1 [33]. There may be some mechanisms, from the perspective of single molecules, for maintaining cellular homeostasis.

Augmented expression of integrins have has been shown to correlate with invasive potential and poor prognosis in many cancers, as integrins contribute to the initial establishment and progression of solid tumors [12,34]. By immunohistochemical detection of the expression of CD98, β1-integrin-A and Ki-67 in human HCC specimens, we found that the expression levels of these molecules were upregulated and correlated with cancer progression and malignancy in HCC patients (Figure 5). CD98, as a β1-integrin activator, could be pursued as both a potential diagnostic marker and an effective anti-cancer target.

Recently, a high-affinity and high-specificity peptide ligand, LXY30, was used for in vivo targeting of α3-integrin-expressing human tumors [35]. Previously, a tripeptidic RGD integrin-recognition motif was able to block tumor invasion and angiogenesis [36,37]. Our work showed that by suppressing β1-integrin activation, exogenous CD98-ICD inhibits the progression of HCC in vitro and in vivo.

## 4. Experimental Section

### 4.1. Cell Culture and Antibodies

Human SMMC-7721 HCC cells were purchased from the Institute of Biochemistry and Cell Biology (IBCB, Shanghai, China). Huh-7 cells were obtained from the Japanese Collection of Research Bioresources. SMMC-7721 and Huh-7 cells were cultured in RPMI1640 (Gibco, New York, NY, USA) with 10% fetal bovine serum, 2 mM glutamine, 100 U/mL penicillin, and 100 µg/mL streptomycin in a 5% CO_2_ atmosphere at 37 °C.

The CD98 antibody for immunohistochemistry and indirect flow cytometry (FACS) was purchased from Abcam (ab23495, Cambridge, UK), and rabbit polyclonal antibodies to CD98 for Western blot and immunofluorescence were from Proteintech, Wuhan, China (15193-1-AP). Anti-CD98-PE (556077) for FACS and anti-FAK (610087) were from BD Bioscience (San Jose, CA, USA). Antibodies to β1-integrin and activated β1-integrin were from Abcam (ab3167) and Millipore Co. (mab2079Z, Merck Millipore, Darmstadt, Germany), respectively. Antibodies against pFAK (3283) and Ki-67 (ab16667) were from CST (Santa Cruz, CA, USA) and Abcam, respectively. Antibody against GFP (sc-9996) was from Santa Cruz Biotechnology (Dallas, TX, USA). Antibody against HA tag (51064-2-AP) was from Proteintech (Wuhan, China). Antibodies against α-tubulin and CD147 (HAb18, IgG1) were developed as previously reported [21,38].

### 4.2. Plasmids

The following plasmids were used: peGFP-N1 (Clontech, Mountain View, CA, USA) and pcDNA3.1 (Invitrogen, Carlsbad, CA, USA). Sequences encoding the signal peptide and intracellular domain of CD98 (NM_002394.5) were cloned into peGFP-N1 (Clontech) with *Nhe* I/*Xho* I to generate CD98-ICD-EGFP. The sequence encoding for amino acids 486–728 of human hook1 (NM_015888.4) was fused with an HA tag by PCR and cloned into the mammalian expression vector pcDNA3.1 to create HK1-S (the cargo-tethering portion of hook1). Mutated ARF6 (Q67L) cloned from pcDNA3.1-ARF6Q67L was inserted into peGFP-N1 with *Nhe* I/*Xho* I to generate ARF6Q67L-EGFP. Cells were transfected with plasmids using Lipofectamine 2000 (Invitrogen) following the manufacturer’s protocols.

### 4.3. Cell Spreading Assay

The cell spreading assay was performed as described previously [21]. Briefly, SMMC-7721 or Huh-7 cells were first transfected with peGFP-N1 or CD98-ICD-EGFP using Lipofectamine 2000 (Invitrogen) for 48 h, and the transfected cells were sorted for GFP fluorescence by means of flow cytometry. Sorted cells were cultured in RPMI1640 with 10% fetal bovine serum and 1 mg/mL G418 (345811, Calbiotech, Shanghai, China). Then, cells (5 × 10^4^ cells/cm^2^) were plated onto 1% Matrigel (BD Bioscience, Franklin Lakes, NJ, USA)-coated glass coverslips for 2 h. Then, the cells were fixed with 4% formaldehyde for 10 min, stained with rhodamine-phalloidin (R415, Invitrogen) and DAPI (Vector laboratories, Burlingame, CA, USA), and viewed using phase microscopy (Olympus, Tokyo, Japan). The cells were assessed with Image J software (1.47v, Wayne Rasband NIH, Bethesda, MD, USA).

### 4.4. Adhesion Assay

The adhesion assay was performed as described previously [39]. Briefly, 96-well cell culture plates were first coated with 50 µL of μL Matrigel (5 mg/mL) in serum-free RPMI 1640 media and incubated at 4 °C overnight. Then the unbound Matrigel was removed with PBS, and the wells were incubated with 2% BSA at 37 °C for 30 min the next day. Then, 2 × 10^4^ of the transfected cells described above were then suspended in serum-free RPMI 1640 media containing 0.1% BSA and seeded into each Matrigel-coated well in triplicate and incubated at 37 °C for 1 h. The plates were then gently washed twice with 200 µL of PBS to remove unbound cells, and stained with 0.2% crystal violet for 10 min, and gently washed with tap water, and dried in air for a day and read at 540 nm using an ELISA reader (Epoch, BioTek, Winooski, VT, USA).

### 4.5. Cell Proliferation Assay

Cell proliferation was quantitatively assessed by a WST-1 cell viability assay according to the manufacturer’s protocols (C0035, Beyotime, Shanghai, China). The cell proliferation rate was measured as the fold change of cells proliferation relative to the control (0 h) and calculated as follows: fold change of cells proliferation rate = ODT/ODC, where ODT is the average OD value of cells at different time points, and ODC is the average OD value of the control samples.

### 4.6. Xenograft Model

SMMC-7721 cells were transfected with CD98-ICD-EGFP or peGFP-N1 (vehicle) and selected with 5 μg/mL of G418 for two weeks to create ICD or vehicle cell lines. Assessment of tumorigenicity in BALB/c nude mice was performed as previously described [21]. Tumor tissues were sectioned and stained with hematoxylin-eosin (H&E), and Ki-67 was used as a mitotic marker. Animal welfare and experimental procedures were performed according to the NIH Guide for the Care and Use of Laboratory Animals.

### 4.7. FACS

FACS was performed as described previously [40]. Briefly, administrated cells were detached and incubated with CD98-PE (1:200, BD) at 4 °C in the dark for 30 min and then analyzed with a FACS Arial I analyzer (BD Biosciences, San Jose, CA, USA) with FCS Express Version 3 software (BD Biosciences, San Jose, CA, USA).

### 4.8. Image Analysis

#### 4.8.1. Immunofluorescence

Immunofluorescence was performed as described previously [21]. Briefly, cells transfected with ARF6Q67L-EGFP, CD98-ICD-EGFP or peGFP-N1 were cultured in a 24-well plate pre-coated with Matrigel. After cells were fixed, permeabilized and blocked. The cells were incubated with a primary antibody (CD98) and Dylight 488 or Dylight 594 labeled secondary antibodies. The nuclei were counterstained with DAPI. The samples were visualized with a confocal microscope using Nikon NIS-Elements software (Nikon, Tokyo, Japan).

#### 4.8.2. Co-Localization Analysis

Co-localization data were evaluated in original images obtained by confocal microscopy. Analysis was performed with Nikon NIS-Elements software (Nikon, Tokyo, Japan).

### 4.9. Co-IP and Western Blot

CD98-ICD-EGFP or EGFP was immunoprecipitated from cell lysates (5 × 10^6^ cells) with 0.5 mg GFP antibody overnight at 4 °C. Immune complexes were captured with protein A/G-Sepharose, and washed immunoprecipitates were resolved by SDS-PAGE and blotted for GFP or β1-integrin. Western blot was performed as described previously [21].

### 4.10. Immunohistochemistry

The immunostaining technique was conducted as described previously [41]. The intensity of staining was scored as 0 (background of negative controls), 1 (weak), 2 (medium) or 3 (strong). The extent of staining was based on an estimate of the number of cells in the whole tumor section at 400× magnification and scored as 0 (0% of cells stained), 1 (1%–25%), 2 (26%–50%), 3 (51%–75%) or 4 (76%–100%). The scores of each tumor sample were calculated by multiplying the intensity staining score by extent staining score to give a final score of 0–12, and the tumors were finally determined as negative (−), score 0; lower expression (+), score ≤ 4; moderate expression (++), score 5–8; and high expression (+++), score ≥ 9. In this study, we grouped all of the samples into high expression group (++ or +++) and the low expression group (− or +) according to the protein expression. Two of the pathologists, without prior knowledge of the clinical data, independently graded the staining intensity in all cases.

### 4.11. Clinical Specimens

The study was approved by the Ethics Committee of the Fourth Military Medical University (FMMU, KY20163014-1, 5 March 2016). A total of 68 pairs of HCC specimens and adjacent non-cancerous specimens were collected from patients who had underwent resection at Xijing Hospital Affiliated with FMMU, with written informed consent of patients. None of the patients underwent chemotherapy or other adjuvant treatments before surgery.

### 4.12. Statistical Analysis

All data were expressed as the mean ± standard deviation (SD), and then processed using GraphPad Prism v5.0 software (GraphPad Software, La Jolla, CA, USA). A Student’s *t*-test was performed to compare the differences between treated groups relative to their paired controls. Tests for association between immunohistochemical expression and clinical pathologic variables were computed using X^2^-test or Fisher’s exact test. Pearson correlation coefficient was used to measure the strength of the association between CD98, β1-integrin-A and Ki-67 expression levels. Values of *p* < 0.05 were considered significant.

## 5. Conclusions

To conclude, our preliminary study indicates that exogenous CD98-ICD has an inhibitory role in HCC malignant development, and CD98-ICD acts as a dominant negative mutant of CD98 that attenuates β1-integrin activation. CD98-ICD may emerge as a promising candidate target for future antitumor treatments.
